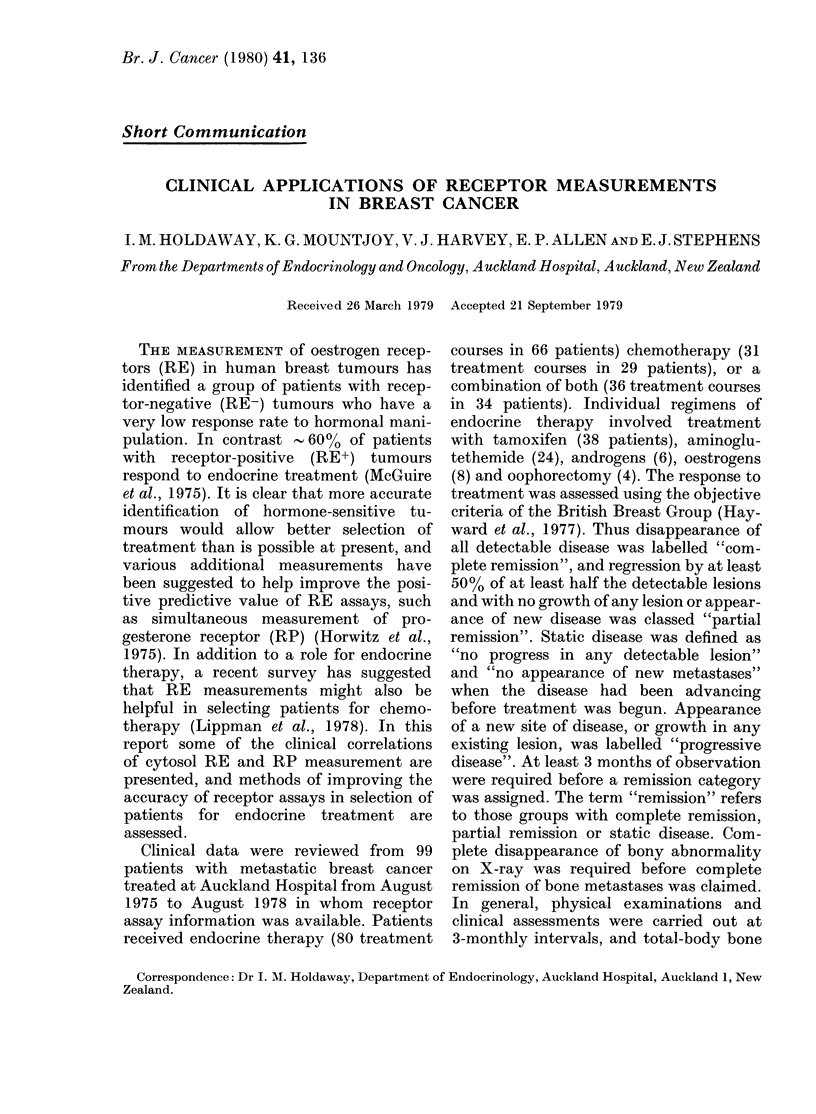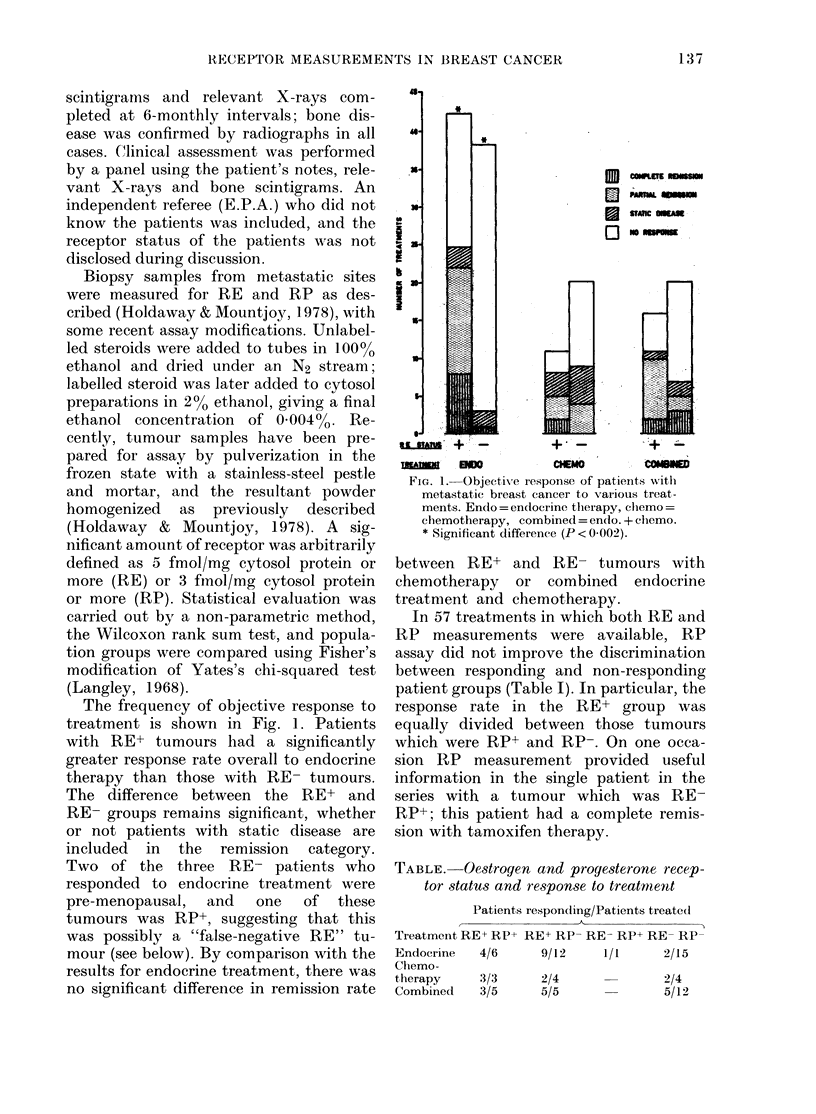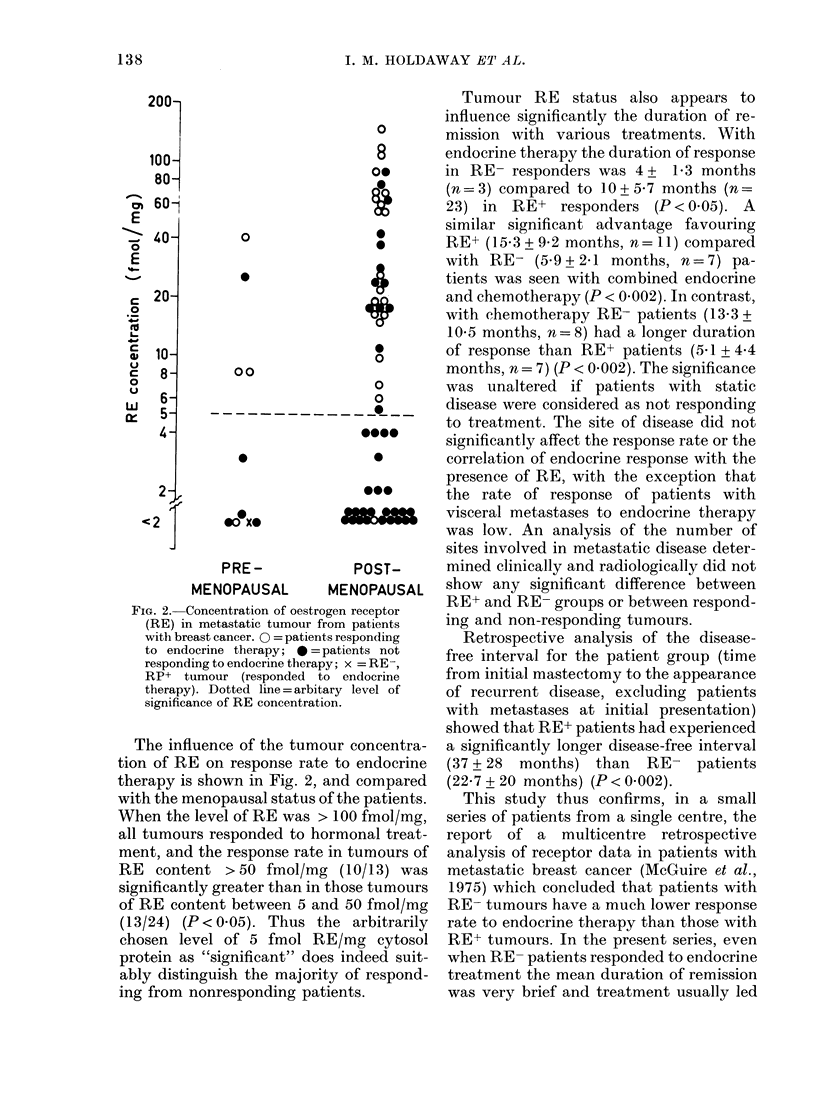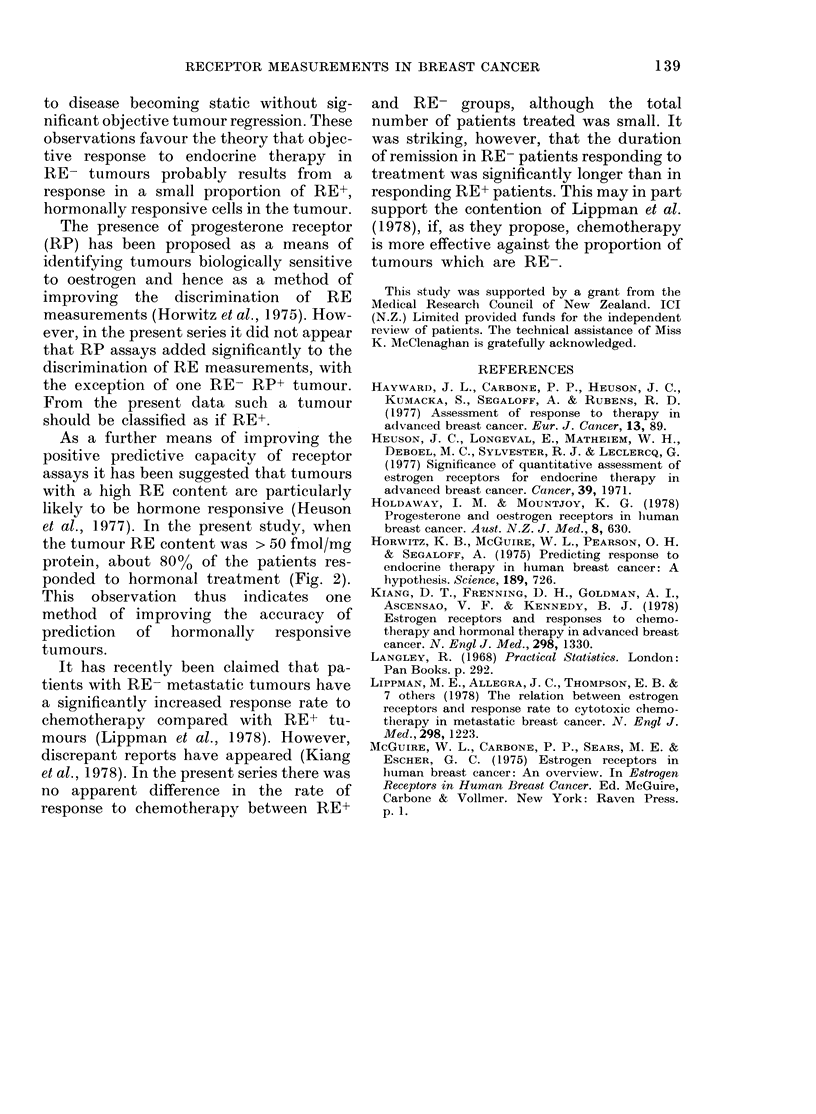# Clinical applications of receptor measurements in breast cancer.

**DOI:** 10.1038/bjc.1980.16

**Published:** 1980-01

**Authors:** I. M. Holdaway, K. G. Mountjoy, V. J. Harvey, E. P. Allen, E. J. Stephens


*Br. J. Cancer* (1980) **41**, 136

***Short Communication***

### CLINICAL APPLICATIONS OF RECEPTOR MEASUREMENTS IN BREAST CANCER

I. M. HOLDAWAY, K. G. MOUNTJOY, V. J. HARVEY, E. P. ALLEN AND E. J. STEPHENS

*From the Departments of Endocrinology and Oncology, Auckland Hospital, Auckland, New Zealand*

Received 26 March 1979 Accepted 21 September 1979

THE MEASUREMENT of oestrogen receptors (RE) in human breast tumours has identified a group of patients with receptor-negative ($RE^-$) tumours who have a very low response rate to hormonal manipulation. In contrast ~60% of patients with receptor-positive ($RE^+$) tumours respond to endocrine treatment (McGuire *et al.*, 1975). It is clear that more accurate identification of hormone-sensitive tumours would allow better selection of treatment than is possible at present, and various additional measurements have been suggested to help improve the positive predictive value of RE assays, such as simultaneous measurement of progesterone receptor (RP) (Horwitz *et al.*, 1975). In addition to a role for endocrine therapy, a recent survey has suggested that RE measurements might also be helpful in selecting patients for chemotherapy (Lippman *et al.*, 1978). In this report some of the clinical correlations of cytosol RE and RP measurement are presented, and methods of improving the accuracy of receptor assays in selection of patients for endocrine treatment are assessed.

Clinical data were reviewed from 99 patients with metastatic breast cancer treated at Auckland Hospital from August 1975 to August 1978 in whom receptor assay information was available. Patients received endocrine therapy (80 treatment courses in 66 patients) chemotherapy (31 treatment courses in 29 patients), or a combination of both (36 treatment courses in 34 patients). Individual regimens of endocrine therapy involved treatment with tamoxifen (38 patients), aminoglutethemide (24), androgens (6), oestrogens (8) and oophorectomy (4). The response to treatment was assessed using the objective criteria of the British Breast Group (Hayward *et al.*, 1977). Thus disappearance of all detectable disease was labelled "complete remission", and regression by at least 50% of at least half the detectable lesions and with no growth of any lesion or appearance of new disease was classed "partial remission". Static disease was defined as "no progress in any detectable lesion" and "no appearance of new metastases" when the disease had been advancing before treatment was begun. Appearance of a new site of disease, or growth in any existing lesion, was labelled "progressive disease". At least 3 months of observation were required before a remission category was assigned. The term "remission" refers to those groups with complete remission, partial remission or static disease. Complete disappearance of bony abnormality on X-ray was required before complete remission of bone metastases was claimed. In general, physical examinations and clinical assessments were carried out at 3-monthly intervals, and total-body bone

Correspondence: Dr I. M. Holdaway, Department of Endocrinology, Auckland Hospital, Auckland 1, New Zealand.

scintigrams and relevant X-rays completed at 6-monthly intervals; bone disease was confirmed by radiographs in all cases. Clinical assessment was performed by a panel using the patient's notes, relevant X-rays and bone scintigrams. An independent referee (E.P.A.) who did not know the patients was included, and the receptor status of the patients was not disclosed during discussion.

Biopsy samples from metastatic sites were measured for RE and RP as described (Holdaway & Mountjoy, 1978), with some recent assay modifications. Unlabelled steroids were added to tubes in 100% ethanol and dried under an $N_2$ stream; labelled steroid was later added to cytosol preparations in 2% ethanol, giving a final ethanol concentration of 0·004%. Recently, tumour samples have been prepared for assay by pulverization in the frozen state with a stainless-steel pestle and mortar, and the resultant powder homogenized as previously described (Holdaway & Mountjoy, 1978). A significant amount of receptor was arbitrarily defined as 5 fmol/mg cytosol protein or more (RE) or 3 fmol/mg cytosol protein or more (RP). Statistical evaluation was carried out by a non-parametric method, the Wilcoxon rank sum test, and population groups were compared using Fisher's modification of Yates's chi-squared test (Langley, 1968).

The frequency of objective response to treatment is shown in Fig. 1. Patients with $RE^+$ tumours had a significantly greater response rate overall to endocrine therapy than those with $RE^-$ tumours. The difference between the $RE^+$ and $RE^-$ groups remains significant, whether or not patients with static disease are included in the remission category. Two of the three $RE^-$ patients who responded to endocrine treatment were pre-menopausal, and one of these tumours was $RP^+$, suggesting that this was possibly a "false-negative RE" tumour (see below). By comparison with the results for endocrine treatment, there was no significant difference in remission rate

Fig. 1.—Objective response of patients with metastatic breast cancer to various treatments. Endo = endocrine therapy, chemo = chemotherapy, combined = endo. + chemo. * Significant difference ($P < 0{\cdot}002$).

between $RE^+$ and $RE^-$ tumours with chemotherapy or combined endocrine treatment and chemotherapy.

In 57 treatments in which both RE and RP measurements were available, RP assay did not improve the discrimination between responding and non-responding patient groups (Table I). In particular, the response rate in the $RE^+$ group was equally divided between those tumours which were $RP^+$ and $RP^-$. On one occasion RP measurement provided useful information in the single patient in the series with a tumour which was $RE^-$ $RP^+$; this patient had a complete remission with tamoxifen therapy.

Table.—*Oestrogen and progesterone receptor status and response to treatment*

| | Patients responding/Patients treated | | | |
|---|---|---|---|---|
| Treatment | $RE^+$ $RP^+$ | $RE^+$ $RP^-$ | $RE^-$ $RP^+$ | $RE^-$ $RP^-$ |
| Endocrine | 4/6 | 9/12 | 1/1 | 2/15 |
| Chemotherapy | 3/3 | 2/4 | — | 2/4 |
| Combined | 3/5 | 5/5 | — | 5/12 |

FIG. 2.—Concentration of oestrogen receptor (RE) in metastatic tumour from patients with breast cancer. ○ = patients responding to endocrine therapy; ● = patients not responding to endocrine therapy; × = $RE^-$, $RP^+$ tumour (responded to endocrine therapy). Dotted line = arbitary level of significance of RE concentration.

The influence of the tumour concentration of RE on response rate to endocrine therapy is shown in Fig. 2, and compared with the menopausal status of the patients. When the level of RE was > 100 fmol/mg, all tumours responded to hormonal treatment, and the response rate in tumours of RE content > 50 fmol/mg (10/13) was significantly greater than in those tumours of RE content between 5 and 50 fmol/mg (13/24) ($P < 0{\cdot}05$). Thus the arbitrarily chosen level of 5 fmol RE/mg cytosol protein as "significant" does indeed suitably distinguish the majority of responding from nonresponding patients.

Tumour RE status also appears to influence significantly the duration of remission with various treatments. With endocrine therapy the duration of response in $RE^-$ responders was $4 \pm 1{\cdot}3$ months ($n = 3$) compared to $10 \pm 5{\cdot}7$ months ($n = 23$) in $RE^+$ responders ($P < 0{\cdot}05$). A similar significant advantage favouring $RE^+$ ($15{\cdot}3 \pm 9{\cdot}2$ months, $n = 11$) compared with $RE^-$ ($5{\cdot}9 \pm 2{\cdot}1$ months, $n = 7$) patients was seen with combined endocrine and chemotherapy ($P < 0{\cdot}002$). In contrast, with chemotherapy $RE^-$ patients ($13{\cdot}3 \pm 10{\cdot}5$ months, $n = 8$) had a longer duration of response than $RE^+$ patients ($5{\cdot}1 \pm 4{\cdot}4$ months, $n = 7$) ($P < 0{\cdot}002$). The significance was unaltered if patients with static disease were considered as not responding to treatment. The site of disease did not significantly affect the response rate or the correlation of endocrine response with the presence of RE, with the exception that the rate of response of patients with visceral metastases to endocrine therapy was low. An analysis of the number of sites involved in metastatic disease determined clinically and radiologically did not show any significant difference between $RE^+$ and $RE^-$ groups or between responding and non-responding tumours.

Retrospective analysis of the disease-free interval for the patient group (time from initial mastectomy to the appearance of recurrent disease, excluding patients with metastases at initial presentation) showed that $RE^+$ patients had experienced a significantly longer disease-free interval ($37 \pm 28$ months) than $RE^-$ patients ($22{\cdot}7 \pm 20$ months) ($P < 0{\cdot}002$).

This study thus confirms, in a small series of patients from a single centre, the report of a multicentre retrospective analysis of receptor data in patients with metastatic breast cancer (McGuire *et al.*, 1975) which concluded that patients with $RE^-$ tumours have a much lower response rate to endocrine therapy than those with $RE^+$ tumours. In the present series, even when $RE^-$ patients responded to endocrine treatment the mean duration of remission was very brief and treatment usually led to disease becoming static without significant objective tumour regression. These observations favour the theory that objective response to endocrine therapy in $RE^-$ tumours probably results from a response in a small proportion of $RE^+$, hormonally responsive cells in the tumour.

The presence of progesterone receptor (RP) has been proposed as a means of identifying tumours biologically sensitive to oestrogen and hence as a method of improving the discrimination of RE measurements (Horwitz *et al.*, 1975). However, in the present series it did not appear that RP assays added significantly to the discrimination of RE measurements, with the exception of one $RE^-$ $RP^+$ tumour. From the present data such a tumour should be classified as if $RE^+$.

As a further means of improving the positive predictive capacity of receptor assays it has been suggested that tumours with a high RE content are particularly likely to be hormone responsive (Heuson *et al.*, 1977). In the present study, when the tumour RE content was $> 50$ fmol/mg protein, about 80% of the patients responded to hormonal treatment (Fig. 2). This observation thus indicates one method of improving the accuracy of prediction of hormonally responsive tumours.

It has recently been claimed that patients with $RE^-$ metastatic tumours have a significantly increased response rate to chemotherapy compared with $RE^+$ tumours (Lippman *et al.*, 1978). However, discrepant reports have appeared (Kiang *et al.*, 1978). In the present series there was no apparent difference in the rate of response to chemotherapy between $RE^+$ and $RE^-$ groups, although the total number of patients treated was small. It was striking, however, that the duration of remission in $RE^-$ patients responding to treatment was significantly longer than in responding $RE^+$ patients. This may in part support the contention of Lippman *et al.* (1978), if, as they propose, chemotherapy is more effective against the proportion of tumours which are $RE^-$.

This study was supported by a grant from the Medical Research Council of New Zealand. ICI (N.Z.) Limited provided funds for the independent review of patients. The technical assistance of Miss K. McClenaghan is gratefully acknowledged.

### REFERENCES

HAYWARD, J. L., CARBONE, P. P., HEUSON, J. C., KUMACKA, S., SEGALOFF, A. & RUBENS, R. D. (1977) Assessment of response to therapy in advanced breast cancer. *Eur. J. Cancer*, **13**, 89.

HEUSON, J. C., LONGEVAL, E., MATHEIEM, W. H., DEBOEL, M. C., SYLVESTER, R. J. & LECLERCQ, G. (1977) Significance of quantitative assessment of estrogen receptors for endocrine therapy in advanced breast cancer. *Cancer*, **39**, 1971.

HOLDAWAY, I. M. & MOUNTJOY, K. G. (1978) Progesterone and oestrogen receptors in human breast cancer. *Aust. N.Z. J. Med.*, **8**, 630.

HORWITZ, K. B., MCGUIRE, W. L., PEARSON, O. H. & SEGALOFF, A. (1975) Predicting response to endocrine therapy in human breast cancer: A hypothesis. *Science*, **189**, 726.

KIANG, D. T., FRENNING, D. H., GOLDMAN, A. I., ASCENSAO, V. F. & KENNEDY, B. J. (1978) Estrogen receptors and responses to chemotherapy and hormonal therapy in advanced breast cancer. *N. Engl J. Med.*, **298**, 1330.

LANGLEY, R. (1968) *Practical Statistics*. London: Pan Books. p. 292.

LIPPMAN, M. E., ALLEGRA, J. C., THOMPSON, E. B. & 7 others (1978) The relation between estrogen receptors and response rate to cytotoxic chemotherapy in metastatic breast cancer. *N. Engl J. Med.*, **298**, 1223.

MCGUIRE, W. L., CARBONE, P. P., SEARS, M. E. & ESCHER, G. C. (1975) Estrogen receptors in human breast cancer: An overview. In *Estrogen Receptors in Human Breast Cancer*. Ed. McGuire, Carbone & Vollmer. New York: Raven Press. p. 1.